# Intérêt de la thérapie antivirale par voie orale dans le traitement des nécroses rétiniennes aigues

**DOI:** 10.11604/pamj.2016.24.169.9972

**Published:** 2016-06-29

**Authors:** Samia El Haouzi, Amina Jait, Omar Lezrek, Tachfouti Samira, Laghmari Amina, Ouafa Cherkaoui, Karman Abdellouahed, Rajae Daoudi

**Affiliations:** 1 Service d’Ophtalmologie A, Hôpital des Spécialités de Rabat, Maroc

**Keywords:** ARN syndrome, perte visuelle, thérapie orale, ARN syndrome, visual loss, oral therapy

## Abstract

Le syndrome de nécrose rétinienne aiguë (RNA) est un syndrome uvéitique rare mais dévastateur pour la vue (pronostic visuel ++). Son diagnostic doit être précoce du fait de sa gravité et du risque de bilatéralisation. C’est une entité rare causée par les virus du groupe Herpès. Chez les patients immunodéprimés, les complications de l’ARN syndrome conduisent souvent à une perte d’acuité visuelle. La confirmation de ce diagnostic dès la découverte de la maladie par la réaction de polymérase en chaîne (PCR) et par le coefficient de charge immunitaire (CCI) le plus souvent par ponction d’humeur aqueuse permet d’optimiser la prise en charge en diminuant le temps nécessaire à une confirmation diagnostique. L’ARN syndrome est de très mauvais pronostic spontané. Des études récentes ont montré que la thérapie orale antivirale (valaciclovir, famciclovir et valganciclovir) et intravitréenne sans traitement intraveineux initial est un traitement efficace de l’ARN. Nous présentons ici le tableau clinique d’un jeune patient âgé de 39 ans admis aux urgences pour baisse d’acuité visuelle. L’examen ophtalmologique a objectivé une nécrose rétinienne aigue unilatérale. Il fut traité par une thérapie antivirale orale (valaciclovir) associée à une corticothérapie et l’évolution était très favorable. Le pronostic de la nécrose rétinienne aiguë ou ARN syndrome est le plus souvent sévère. Le traitement de cette affection doit être le plus précoce possible afin de limiter une bilatéralisation et la survenue des complications. Cette observation confirme que la thérapie orale antivirale (valaciclovir, famciclovir et valganciclovir) sans traitement intraveineux initial est un traitement efficace de l’ARN.

## Abstract (en)

Acute retinal necrosis syndrome (ARNS) is a rare but devastating uveitic syndrome with devastating visual outcome (visual prognosis ++). It should be diagnosed as early as possible because of its severity and its risk of bilateralization. This is a rare entity caused by the group of herpes viruses. In immunocompromised patients the complications of RNA syndrome often lead to loss of visual acuity. After the discovery of this disease using polymerase chain reaction (PCR) and immune load coefficient (ILC) usually by puncturing the cornea to evacuate the aqueous humour, the confirmation of this diagnosis allows for faster optimization of disease management decreasing the time required to confirm the diagnosis. ARN syndrome is associated with a very poor prognosis. Recent studies have shown that oral antiviral (valaciclovir, famciclovir and valganciclovir) and intravitreal therapies without initial intravenous treatment are an effective treatment for RNA. Observation: We report the clinical features of a 39-year-old young patient admitted to our emergency department with visual loss. Ophthalmologic examination objectified unilateral acute retinal necrosis. The patient was treated with oral antiviral therapy (valacyclovir) associated with corticosteroids and evolution was very favorable. Conclusion: the prognosis of acute retinal necrosis syndrome or RNA is usually severe. The patient should be treated as early as possible in order to limit bilateralization and the occurrence of complications. This study confirms that oral antiviral (valacyclovir, famciclovir and valganciclovir) and intravitreal therapies without initial intravenous treatment are an effective treatment for RNA.

## Introduction

La nécrose rétinienne aiguë ou ARN syndrome est une affection rare d’origine virale et dont le pronostic est le plus souvent sévère. Elle se caractérise par une inflammation oculaire marquée, accompagnée d’une nécrose rétinienne d’extension progressive, de vascularites rétiniennes et parfois d’atteintes extra oculaires. C’est une entité rare causée par les virus du groupe Herpès. La confirmation du diagnostic clinique, souvent par ponction d’humeur aqueuse, le plus tôt possible permet d’optimiser la prise en charge. Les buts du traitement visent à accélérer la résolution de l’infection dans l’œil infecté et de prévenir l’atteinte de l’œil controlatéral.

## Patient et observation

**Figure 1:**
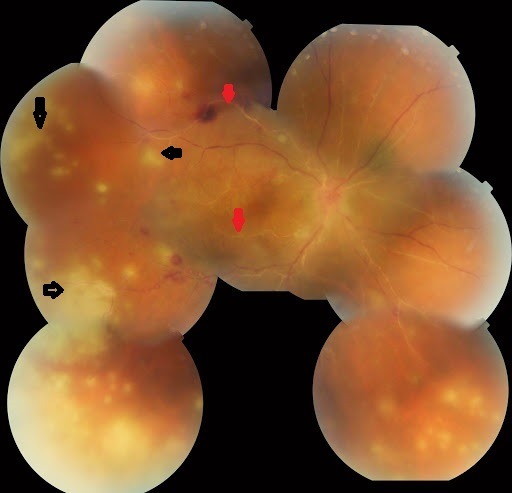
Hyalite visible au niveau de la rétinophotographie couleur du pôle postérieur. En périphérie inférieure et temporale, des zones de nécrose rétinienne jaunes sont visibles (flèches noires) et les vascularites rétiniennes (flèches rouges)

**Figure 2:**
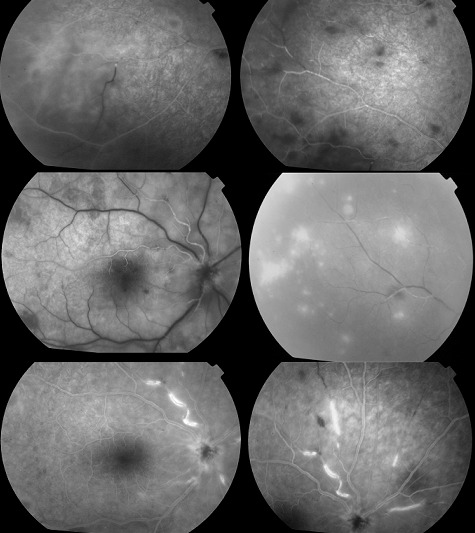
Angiographie à la Fluorescéine montrant des zones d’ischémie rétinienne étendue, des plages de nécrose rétinienne et des signes de vascularite avec diffusion papillaire

**Figure 3:**
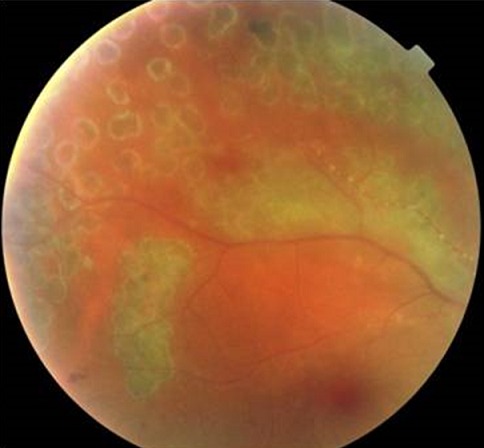
Rétinophotographie montrant les impacts de laser prophylactique

**Figure 4:**
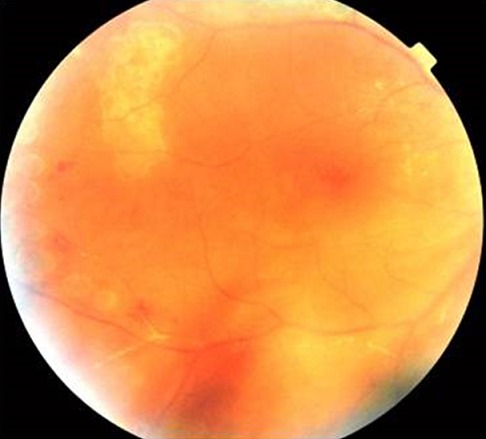
Rétinophotographie montrant la régression des foyers de nécrose

Homme de 39 ans, emmétrope et sans antécédents particulier; s’est présenté aux urgences pour baisse d’acuité visuelle brutale unilatérale OD; l’examen ophtalmologique trouvait une acuité visuelle réduite à 2/10 de l’œil droit et de 10/10 de l’œil gauche, les pressions oculaire correspondantes étaient de 20 mmHg et de 12 mmhg L’examen biomicroscopique de l’œil droit a révélé au niveau du segment antérieur des précipités rétro-cornéens fins pigmentés inférieurs et un Tyndall cellulaire de chambre antérieure 2 croix. Le Fond d´œil a objectivé un Tyndall vitréen 3 crois, des foyers de nécrose rétinienne blanchâtres s’étendant de la périphérie au centre avec artérite occlusive marquée et œdème papillaire (Figure 1). Les Segments antérieur et postérieur de l’œil gauche étaient normaux. Angiographie à la fluorescéine de l’OD a montré un retard de remplissage artériel, avec zone d’ischémie rétinienne étendue (Figure 2). Les diagnostics de nécrose rétinienne aiguë et de rétinite à cytomégalovirus (ne connaissant pas le statut immunitaire récent du patient) sont immédiatement évoqués. Un traitement d’attaque fut instauré en urgence, sans attendre les résultats des prélèvements réalisés en urgence: sérologies des virus HIV1 et 2, VZV, CMV, HSV1 et 2, ponction lombaire et recherche dans le liquide céphalorachidien. Le traitement a associé une chimiothérapie antivirale par le valaciclovir oral; à raison de 1g 3 fois par jour pendant 6 semaines et une corticothérapie orale: 1 mg/kg de Prednisone débutée 48h après le début de traitment antiviral. Un traitement topique à base de corticoïde et de cycloplégique a été préconisé pour éviter les synéchies irido-cristallinienne. Une ponction de chambre antérieure est réalisée. Nous avons finalement confirmé l’atteinte herpétique. Le virus HSV2 est retrouvé par PCR dans l’humeur aqueuse; les sérologies HIV sont négatives et le rapport CD3/CD4 normal. Un Laser prophylactique a été réalisé après les 2 premières semaines pour prévenir un décollement de rétine (Figure 3). L’évolution était marquée par une amélioration sous traitement, une acuité visuelle qui a remonté à 12/10, la tension oculaire était de 14 mmhg, une régression de l’œdème papillaire et des foyers de nécrose (Figure 4) de l’œil droit. L’œil gauche toujours sans particularités Le traitement était alors relayé par un traitement d’entretien antiviral pendant 5 mois (500 mg 3 fois par jour) avec dégression progressive de la corticothérapie.

## Discussion

L’ARN syndrome est un syndrome uvéitique rare mais dévastateur pour la vue. Il a été rapporté pour la première fois par Urayama et al.en 1971 [1]. Dans leur série de 6 patients, les auteurs ont décrit la triade: panuvéite, vascularite rétinienne, et de larges zones de nécrose rétinienne. En 1982, Culbertson et al. [2] ont montré la présence de particules d’Herpès virus en microscopie électronique dans un œil énuclé atteint de l’ARN syndrome. En 1986, ils étaient capables de cultiver le VZV depuis un œil atteint. Les critères de diagnostic standardisés de l’American Uveitis Society [3] sont: un ou plusieurs foyers de nécrose rétinienne à limites distinctes; une progression de la maladie en l’absence de traitement; la présence d’une vasculopathie occlusive; une réaction inflammatoire dans le vitré ou dans la cavité vitréenne.

Son diagnostic reste difficile lors de la phase précoce. Les signes fonctionnels sont variables. L’acuité visuelle est en général conservée à la phase précoce de l’atteinte car la nécrose épargne encore le pôle postérieur et les décollements de rétine sont rares au stade initial. Le segment antérieur est en général le siège d’une inflammation importante plus ou moins associée à une hypertonie oculaire; le vitré est également très inflammatoire contrairement aux rétinites virales de l’immunodéprimé. La nécrose rétinienne se présente initialement en moyenne périphérie rétinienne par des opacités blanchâtres rondes surélevées par rapport à la rétine saine adjacente [4]. Elle progresse en l’absence de traitement de façon circonférentielle et dans le sens antéropostérieur jusqu’à une coalescence sur 360° et une atteinte du pôle postérieur en 2 à 15 jours. Des vascularites rétiniennes prédominant sur le réseau artériel sont responsables d’une aggravation de la nécrose rétinienne [5]. L’ARN est une affection dont l’origine virale est connue depuis une vingtaine d’années (1982) [6, 7]. Plusieurs virus de la famille des herpes viridae peuvent être responsables. En premier lieu, le virus de la varicelle et du zona (VZV) est le virus le plus fréquemment retrouvé dans les ARN syndromes. Les virus herpes simplex (HSV) sont ensuite retrouvés dans une moindre proportion d’ARN, en particulier l’HSV2 moins fréquent que l’HSV1. Les différentes séries de la littérature rapportent une distribution bimodale de l’âge des patients atteints en fonction du virus: les ARN syndromes secondaires au VZV et à l’HSV1 concernent des patients âgés en moyenne de plus de 50 ans tandis que les atteintes dues à l’HSV2 touchent des patients de 20 ans en moyenne [8]. Néanmoins, peu de cas secondaires à l’HSV2 sont rapportés. Le cytomégalovirus (CMV), est, quant à lui, beaucoup plus rarement en cause. La confirmation du diagnostic clinique, souvent par ponction d’humeur aqueuse, fait le plus tôt possible permet d’optimiser la prise en charge. Les buts du traitement visent à accélérer la résolution de l’infection dans l’œil infecté et de prévenir l’atteinte de l’œil controlatérale. L’aciclovir intraveineux a les effets secondaires suivants: augmentation des taux sériques de créatinine, calculs urinaires, élévation des enzymes hépatiques et toxicité au niveau du système nerveux central (léthargie, délire, crises épileptiques). Le traitement standard pour traiter l’ARN a été défini par Palay et al. et consiste en l’aciclovir intraveineux (IV) (500 mg/m^2^ administré 3 fois par jour ou 10 mg/kg toutes les 8 heures) pendant 7 à 10 jours, suivi par de l’aciclovir oral (800 mg 5 fois par jour) pendant 6-12 semaines après le traitement initial Le valaciclovir est un antiviral. C’est l´ester L-valine de l´aciclovir, qui est un analogue nucléosidique purinique (guanine).

Chez l´homme, le valaciclovir est rapidement et presque entièrement métabolisé en aciclovir et en valine, vraisemblablement par l´enzyme appelée l´hydrolase valaciclovir. L´aciclovir est un inhibiteur spécifique de la synthèse de l´ADN viral des herpès-virus, avec une activité in vitro sur les virus Herpes simplex (HSV) de type 1 et de type 2, varicelle-zona (VZV), cytomégalovirus (CMV), Epstein-Barr (EBV) et herpès-virus humain de type 6 (HHV-6). Des études récentes (Aizman A) ont montré que la thérapie orale antivirale (valaciclovir, famciclovir et valganciclovir) et intravitréenne sans traitement intraveineux initial est un traitement efficace de l’ARN [9]. En effet les concentrations sanguines sont similaires à celles obtenues avec administration IV, car ces molécules ont une pharmacocinétique qui leur permet d’atteindre des taux sériques thérapeutiques quand ils sont convertis dans leur forme active. Guex-Crosier et al. ont récemment suggéré qu’une dose plus forte de valaciclovir oral (2 g 4 fois par jour) pourrait représenter une bonne alternative à l’aciclovir intraveineux. Notre observation rejoint ces études et illustre l’intérêt de la voie orale du valaciclovir dans le traitement de RNA sans traitement intraveineux initial, ceci permet d’éviter la toxicité l’aciclovir intraveineux.

## Conclusion

La nécrose rétinienne aiguë est une entité rare causée par les virus du groupe Herpès. Elle doit être évoquée de principe devant toute panuvéite. Elle reste une affection grave malgré les traitements antiviraux en raison du risque de bilatéralisation précoce et des complications rétiniennes. Le valaciclovir par voie orale reste un traitement efficace associée à une corticothérapie et à une surveillance étroite.

## References

[1] Urayama A, Yamada N, Sa Saki T (1971). Unilateral acute uveitis with periarteritis and detachment [in Japanese]. Rinsho Ganka.

[2] Culbertson WW, Blumenkranz MS, Haines H (1982). The acute retinal necrosis syndrome: Part 2, Histopathology and etiology. Ophthalmology.

[3] Holland GN (1994). Standard diagnostic criteria for the acute retinal necrosis syndrome: Executive Committee of the American Uveitis Society. Am J Ophthalmol.

[4] Labetoulle M (1995). Le syndrome de nécrose rétinienne aiguë. J Fr Ophtalmol.

[5] Offret H (2000). Œil et virus. Rapport de la Société Française d’Ophtalmologie.

[6] Culbertson WW, Blummmenkranz MS, Haines H, Gass DM, Mitchell KB, Norton EW (1982). The acute retinal necrosis syndrome: Part II, Histopathology and etiology. Ophthalmology.

[7] Ganatra JB, Chandler D, Santos C, Kuppermann B, Margolis TP (2000). Viral causes of the acute retinal necrosis syndrome. Am J Ophthalmol.

[8] Van Gelder RN, Willig JL, Holland GN, Kaplan HJ (2001). Herpes simplex virus type 2 as a cause of acute retinal necrosis syndrome in young patients. Ophthalmology.

[9] Aizman A, Johnson MW, Elner SG (2007). Treatment of acute retinal necrosis syndrome with oral antiviral medications. Ophthalmology.

